# Supplementary material for: Comparative analysis of surgical outcomes: Video-assisted thoracoscopic surgery versus open thoracotomy in organizing thoracic empyema management
Source: PLoS One. 2025 Oct 9;20(10):e0332618. doi: 10.1371/journal.pone.0332618 (PMC12510550; doi:10.1371/journal.pone.0332618)
Supplement: Attachment — Submitted filename: Response_to_Reviewer_Comments_auresp_2.docx [file pone.0332618.s004.docx]

**Response to Reviewer Comments**

Dear Editors and Reviewers,

We would like to thank you for your careful review and valuable comments on our manuscript, entitled “Comparative Analysis of Surgical Outcomes: Video-Assisted Thoracoscopic Surgery versus Open Thoracotomy in Organizing Thoracic Empyema Management”. We appreciate the time and effort you and the reviewers have dedicated to enhancing the quality of our work.

Please find our responses to each reviewer’s comments below. All changes made in the revised manuscript are highlighted for your convenience.

Reviewer #1:
Thank you for the revisions, which have significantly improved your manuscript. However, there are a few remaining points:

1. I appreciate that you have acknowledged the exclusion of conversion cases, but this remains an important limitation as conversions represent clinically relevant outcomes in real-world practice. A sensitivity analysis including these cases would strengthen the study, but I accept that this may not be feasible with the current dataset.

Response: Thank you for your thoughtful comment and for highlighting the clinical relevance of conversion cases. In line with your suggestion, we have conducted a sensitivity analysis that includes the conversion cases and have incorporated the results into the revised manuscript.

We added the paragraph in the method part as shown below;

“Patients who initially underwent VATS but were converted to OT due to a very thick organized peel covering the visceral pleura, resulting in incomplete decortication, or due to unexpected complications, were excluded from the primary analysis to minimize indication bias (per-protocol approach). However, to reflect real-world practice, a sensitivity analysis that included conversion cases was performed using both intention-to-treat and as-treated analytic approaches.”

We have also corrected the error regarding the conversion cases, which comprised 4.8% of patients, rather than 5 cases as previously stated. In addition, we have revised the Results section and updated Table 3 to reflect this information, as shown below.

“For the intention-to-treat analytic approach, results across all patients were consistent with the primary analysis, with the exception that patients in the VATS group were more likely to experience a reduction in pain scores compared with those in the OT group. However, in the propensity-matched cohort, there was no statistically significant difference in pain scores, although a trend toward greater pain reduction in the VATS group was observed (Table 3). For the as-treated analytic approach, among all patients, the VATS group demonstrated reduced intraoperative blood loss, shorter operative time, and a higher proportion of fully expanded lungs at discharge. In the propensity-matched cohort, differences in intraoperative blood loss and operative time remained significant; however, the difference in the proportion of fully expanded lungs at discharge did not reach statistical significance. Nevertheless, a higher rate of fully expanded lungs at two weeks post-discharge was observed in the VATS group (Table 3).”

**“Table 3.** Multivariable regression analysis for outcome variables comparing between the two groups (VATS versus OT)

| Outcome variables | All patient cohort | | | Propensity-matched cohort | | |
| --- | --- | --- | --- | --- | --- | --- |
|  | Estimate | 95 % CI | p-value | Estimate* | 95 % CI | p-value |
| **Primary analysis (Per-protocol analytic approach)** | | | | | | |
| Intraoperative blood loss (ml) | -35.58^a^ | -141.17, 70.12 | 0.508 | -34.20 | -162.89, 94.49 | 0.601 |
| Operative time (min) | -6.37^a^ | -27.17, 14.44 | 0.548 | 1.25 | -24.42, 26.91 | 0.924 |
| ICU stay (day) | 1.38^a^ | -0.68, 3.44 | 0.189 | 0.68 | -1.56, 3.23 | 0.494 |
| Decreased pain score | 0.71^b^ | -0.04, 1.46 | 0.065 | 0.45 | -0.29, 1.18 | 0.236 |
| Fully expanded lung at discharge date | 1.24^c^ | 1.07, 1.45 | 0.006 | 1.21 | 1.01, 1.45 | 0.038 |
| Fully expanded lung at 2 weeks after discharge | 1.08^c^ | 0.95, 1.23 | 0.221 | 1.14 | 0.97, 1.34 | 0.117 |
| **Intention-to-treat analytic approach** | | | | | | |
| Intraoperative blood loss (ml) | -3.34^a^ | -114.85, 108.16 | 0.953 | -7.42 | -125.53, 110.69 | 0.902 |
| Operative time (min) | -7.47^a^ | -28.57, 13.63 | 0.487 | -4.84 | -27.67, 17.99 | 0.677 |
| ICU stay (day) | 1.38^a^ | -0.58, 3.34 | 0.167 | 1.87 | -0.23, 3.97 | 0.080 |
| Decreased pain score | 0.84^b^ | 0.10, 1.57 | 0.025 | 0.78 | -0.01, 1.56 | 0.051 |
| Fully expanded lung at discharge date | 1.25^c^ | 1.07, 1.46 | 0.004 | 1.20 | 1.03, 1.41 | 0.023 |
| Fully expanded lung at 2 weeks after discharge | 1.07^c^ | 0.94, 1.21 | 0.309 | 1.10 | 0.98, 1.24 | 0.104 |
| **As treated analytic approach** | | | | | | |
| Intraoperative blood loss (ml) | -179.76^a^ | -276.167, -83.36 | <0.001 | -215.29 | -331.08, -99.50 | <0.001 |
| Operative time (min) | -24.85^a^ | -43.25, -6.45 | 0.008 | -23.27 | -43.64, -2.91 | 0.025 |
| ICU stay (day) | 1.24^a^ | -0.48, 2.96 | 0.157 | 1.39 | -0.44, 3.22 | 0.135 |
| Decreased pain score | 0.22^b^ | -0.21, 0.65 | 0.314 | 0.71 | -0.09, 1.50 | 0.083 |
| Fully expanded lung at discharge date | 1.19^c^ | 1.03, 1.38 | 0.016 | 1.20 | 0.99, 1.45 | 0.062 |
| Fully expanded lung at 2 weeks after discharge | 1.07^c^ | 0.95, 1.21 | 0.254 | 1.30 | 1.11, 1.53 | 0.002 |

Intention-to-treat analysis: Patients who underwent conversion from VATS to open thoracotomy were included in the VATS group.

As-treated analysis: Patients who were converted from VATS to open thoracotomy were included in the open thoracotomy group.

^a^Analyzed by multiple linear regression analysis adjusted by multi-loculation, thick pleural peel, year of surgery, and duration symptoms prior to diagnosis, and presented with mean difference (MD) with 95 % confidence intervals.

^b^Analyzed by repeated measurements method adjusted by age, gender, operative time, year of surgery, and duration symptoms prior to diagnosis.

^c^Analyzed by exponential risk regression analysis adjusted by multi-loculation, thick pleural peel, year of surgery, and duration symptoms prior to diagnosis, and presented with risk ratio (RR) with 95 % confidence intervals.

*Adjusted for year of surgery and duration symptoms prior to diagnosis.”

We added and delete paragraph in discussion part as shown below.

“However, to better reflect real-world clinical practice, a sensitivity analysis including conversion cases was performed using both intention-to-treat and as-treated analytic approaches. The results of these analyses continued to demonstrate the benefits of VATS over the OT group.”

“Furthermore, in the as-treated analysis, VATS was associated with reduced intraoperative blood loss, shorter operative time, and a higher proportion of fully expanded lungs at two weeks post-discharge.”

“This finding contrasts with previous studies that have reported shorter operative times with the VATS approach [11]. In the as-treated analytic approach, VATS was associated with shorter operative times; however, it is important to note that conversion cases-often representing more complex cases or those in which complete decortication could not be achieved via VATS approach-were included in the OT group, potentially contributing to the longer operative times observed in this group.”

2. I believe residual confounding is still possible despite propensity matching and multivariable adjustment (e.g., longer duration of symptoms in the thoracotomy group). I suggest highlighting this more clearly in the discussion.

Response: Thank you for your insightful comment regarding the possibility of residual confounding, even after propensity matching and multivariable adjustment. We agree with your assessment that unmeasured confounding factors—such as the longer duration of symptoms in the thoracotomy group—may still influence our findings. In response, we have revised the Discussion section to more explicitly acknowledge this limitation. Specifically, we added the following statement:

“Although propensity score matching and multivariable adjustment were used in this study to minimize confounding, the possibility of residual confounding from unmeasured variables still exists. This limitation should be considered when interpreting the results of this study.”

From previous revised manuscript we already discuss this point as shown in the paragraph below:

“Additionally, despite propensity score matching, patients in the OT group had a significantly longer duration of symptoms, suggesting more advanced disease at the time of surgery. This difference could have influenced clinical outcomes and may, in part, account for the comparable results observed between the OT and VATS groups. Therefore, residual confounding due to differences in baseline disease severity cannot be completely excluded, even though this variable was adjusted for in the multivariable analysis.”

3. There are still typos throughout (e.g., “blood lose” should be “blood loss”).

Response: Thank you for highlighting the remaining typographical errors in our manuscript. We apologize for these oversights. We have carefully proofread the entire document and corrected all identified typos, including changing “blood lose” to “blood loss.” We have also performed a thorough language check to improve clarity and accuracy throughout the manuscript.
