# Supplementary material for: Comparative analysis of surgical outcomes: Video-assisted thoracoscopic surgery versus open thoracotomy in organizing thoracic empyema management
Source: PLoS One. 2025 Oct 9;20(10):e0332618. doi: 10.1371/journal.pone.0332618 (PMC12510550; doi:10.1371/journal.pone.0332618)
Supplement: Attachment — Submitted filename: Response to Reviewer Comments.docx [file pone.0332618.s003.docx]

**Response to Reviewer Comments**

Dear Editors and Reviewers,

We would like to thank you for your careful review and valuable comments on our manuscript, entitled “Comparative Analysis of Surgical Outcomes: Video-Assisted Thoracoscopic Surgery versus Open Thoracotomy in Organizing Thoracic Empyema Management”. We appreciate the time and effort you and the reviewers have dedicated to enhancing the quality of our work.

Please find our responses to each reviewer’s comments below. All changes made in the revised manuscript are highlighted for your convenience.

**Reviewer #1:**

**Comment 1:**
**You mention that VATS was introduced in 2015 and became the preferred technique thereafter. Could you comment on whether this shift over time may have introduced a performance/experience bias (e.g., learning curve, evolving perioperative care), and how this may have impacted the internal validity of your findings?**

**Response:** We have re-analyzed data and revised results (Table 3), add sentences in discussion section to clarify the research gap as follows:

***Table 3.*** *Multivariable regression analysis for outcome variables comparing between the two groups (VATS versus OT)*

| *Outcome variables* | *All patient cohort* | | | *Propensity-matched cohort* | | |
| --- | --- | --- | --- | --- | --- | --- |
|  | *Estimate* | *95 % CI* | *p-value* | *Estimate** | *95 % CI* | *p-value* |
| *Intraoperative blood loss (ml)* | *-35.58^a^* | *-141.17, 70.12* | *0.508* | *-34.20* | *-162.89, 94.49* | *0.601* |
| *Operative time (min)* | *-6.37^a^* | *-27.17, 14.44* | *0.548* | *1.25* | *-24.42, 26.91* | *0.924* |
| *ICU stay (day)* | *1.38^a^* | *-0.68, 3.44* | *0.189* | *0.68* | *-1.56, 3.23* | *0.494* |
| *Decreased pain score* | *0.71^b^* | *-0.04, 1.46* | *0.065* | *0.45* | *-0.29, 1.18* | *0.236* |
| *Fully expanded lung at discharge date* | *1.24^c^* | *1.07, 1.45* | *0.006* | *1.21* | *1.01, 1.45* | *0.038* |
| *Fully expanded lung at 2 weeks after discharge* | *1.08^c^* | *0.95, 1.23* | *0.221* | *1.14* | *0.97, 1.34* | *0.117* |

*^a^Analyzed by multiple linear regression analysis adjusted by multi-loculation, thick pleural peel, year of surgery, and duration symptoms prior to diagnosis, and presented with mean difference (MD) with 95 % confidence intervals.*

*^b^Analyzed by repeated measurements method adjusted by age, gender, operative time, year of surgery, and duration symptoms prior to diagnosis.*

*^c^Analyzed by exponential risk regression analysis adjusted by multi-loculation, thick pleural peel, year of surgery, and duration symptoms prior to diagnosis, and presented with risk ratio (RR) with 95 % confidence intervals.*

**Adjusted for year of surgery and duration symptoms prior to diagnosis.*

*We added sentences in discussion part as shown below;*

*“However, a notable distinction observed in this study, compared to certain previous reports, is the longer operative time associated with VATS in the propensity-matched cohort, although this difference did not reach statistical significance. While the VATS approach initially appeared to require extended operative time for decortication compared to OT,* *increased surgeon experience and progression along the learning curve contributed to improved efficiency over time. After adjustment for operative period in our analysis, no significant difference in operative time was observed between the groups in either the full cohort or the propensity-matched cohort. This finding contrasts with studies suggesting shorter operative times with VATS[9]. The variation in operative time and other outcomes may be attributable to differences in surgical techniques, patient populations, the thickness of organized peels covering lung, or institutional VATS practices [10]”*

We added sentences in limitation of this study as shown below;

*“However, the study has limitations. The retrospective design and non-randomized allocation may introduce bias and limit causal conclusions. Information bias from reliance on medical records is possible. Despite a moderate sample size, the study may lack power for detecting smaller differences, especially in the propensity-matched cohort analysis. The transition from open surgery to VATS is associated with a well-recognized learning curve, and varying levels of surgeon experience may influence surgical outcomes, particularly during the early phase following the adoption of VATS. In addition, perioperative care protocols may have evolved during the study period, possibly affecting patient outcomes. We attempted to minimize these confounding effects by controlling for the year of surgery in statistical analyses.”*

**Comment 2: Surgeon experience may influence operative time and outcomes, especially given the reported differences in timing of VATS adoption. Were the same surgeons performing both techniques? Was surgical expertise considered in the analysis?**

**Response:** In our study, both VATS and open thoracotomy procedures were performed by a consistent team of thoracic surgeons, all of whom had experience with both techniques. However, the adoption of VATS introduced a learning curve which may have impacted operative times and outcomes, particularly in the initial phase following its implementation. To address the potential influence of surgical expertise, we adjusted for time period of surgery in our analysis as shown in comment 1. Nevertheless, we acknowledge that differences in individual experience and progression along the learning curve are potential sources of bias that could not be fully accounted for in our retrospective analysis. This limitation has been noted in the revised manuscript as shown below;

In patients and methods part, we added sentence “*Both VATS and OT procedures were performed by a consistent team of thoracic surgeons, all of whom had experience with both techniques.*”

Table 3 which is the multivariable analysis, we adjusted year of surgery in the multivariable model both in full cohort and propensity-matched cohort and add some paragraph in discussion part as shown in comment 1.

**Comment 3: What tool did you use for pain measurement (e.g., numeric rating scale, visual analogue scale). Please clarify how pain was assessed, who collected the data, and whether assessors were blinded to the surgical approach.**

**Response:** Postoperative pain was assessed using the Visual Analog Scale (VAS). Pain assessments were performed by a dedicated team of well-trained nursing staff who were not involved in the study. These assessments were routinely conducted at the immediate postoperative period, as well as at 8, 24, and 48 hours after surgery, and at the time of discharge. The nursing staff were not formally blinded to the surgical approach due to the nature of clinical care; however, they were not involved in the study design, data analysis, or patient allocation. We have added these details to the Methods section (Outcome Definition) in the revised manuscript as shown below;

“*Pain scores were assessed using the Visual Analog Scale by a team of well-trained nursing staff who were not involved in the study. Assessments were conducted at the immediate postoperative period, as well as at 8, 24, and 48 hours postoperatively, and at the time of discharge*.”

**Comment 4: A large proportion of patients in the open thoracotomy group did not undergo preoperative CT scanning. Could this missing imaging data may have influenced surgical planning?**

**Response:** Due to hospital policy during the study period, preoperative CT scans were not routinely performed for all cases of thoracic empyema. CT imaging was typically reserved for patients deemed to be at higher risk, such as those of advanced age, those with significant comorbidities, or those with suspected underlying pulmonary disease or malignancy. Regardless of preoperative imaging, the surgical objective in all cases was to achieve complete decortication and maximal lung re-expansion. Therefore, the absence of preoperative CT scans is unlikely to have significantly influenced the surgical approach or outcomes. Additionally, while some selection bias may have occurred, propensity score matching was performed, resulting in a balanced proportion of preoperative CT imaging between the groups after matching.

We added this details in result part and discussion part as shown below;

In result part, “*Regarding to preoperative CT scans, the proportion of patients who underwent preoperative CT scanning in the VATS group was higher than that in OT group*.”

In discussion part, “*Regarding to pre-operative CT scan, due to hospital policy during the study period, preoperative CT scans were not routinely performed for all cases of thoracic empyema. CT imaging was typically reserved for patients deemed to be at higher risk, such as those of advanced age, those with significant comorbidities, or those with suspected underlying pulmonary disease or malignancy. Regardless of preoperative imaging, the surgical objective in all cases was to achieve complete decortication and maximal lung re-expansion. Therefore, the absence of preoperative CT scans is unlikely to have significantly influenced the surgical approach or outcomes. Additionally, while some selection bias may have occurred, propensity score matching was performed, resulting in a balanced proportion of preoperative CT imaging between the groups after matching as shown in Table 1.”*

**Comment 5: Could you explain why you excluded patients who were converted from VATS to open thoracotomy, since conversions are part of real-world VATS outcomes?**

**Response:** We excluded patients who were converted from VATS to open thoracotomy to ensure a clear comparison between the intended VATS and open thoracotomy groups, and to minimize confounding variables related to intraoperative decision-making and patient selection. We agree that conversions represent important real-world outcomes of VATS; however, our primary aim was to compare perioperative and postoperative outcomes between patients who completed either approach as initially planned. We acknowledge this as a limitation of our study and have now discussed its potential impact in the revised manuscript as follow;

“*One notable limitation of our study is the exclusion of patients who were converted from VATS to OT during the procedure. While this approach was intended to facilitate a direct comparison between patients who completed either VATS or OT as originally planned, it may have introduced selection bias. Consequently, our findings may not fully reflect the perioperative and postoperative outcomes associated with conversion cases, which represent important real-world events in VATS practice. Therefore, the generalizability of our results to all patients undergoing VATS, particularly those at higher risk for conversion, may be limited. Future studies should consider including converted cases to provide a more comprehensive assessment of surgical outcomes*.”

**Comment 6**: You state that five patients were excluded due to conversion from VATS to open thoracotomy. Could you also include the overall conversion rate?

**Response:** The overall conversion rate from VATS to open thoracotomy in our study was 2.5 %, as five out of 204 patients required conversion. We have now included this information in the revised manuscript at result part for clarity as follow;

*“Five patients were excluded from this study due to conversion from VATS to OT, resulting in a conversion rate of 2.5 % (5 out of 204 patients).”*

**Comment 7. Could you define “complete decortication”? Was it based on the surgeon’s judgment, or did you apply standardized criteria across all cases?**

**Response:** In our study, “complete decortication” was defined as the full removal of the fibrous peel from both the visceral and parietal pleura to allow complete lung re-expansion, as confirmed intraoperatively. This assessment was primarily based on the surgeon’s judgment during the procedure. We acknowledge the subjective nature of this definition, as no universally standardized criteria were formally applied across all cases. This clarification has been included in the revised manuscript at the outcome definition part as follow;

“*Complete decortication was defined as the meticulous removal of the organized fibrous peel from visceral pleural surfaces to achieve full lung re-expansion. The extent of decortication was confirmed intraoperatively by the operating surgeon through direct inspection and assessment of pulmonary re-expansion. This evaluation was based on intraoperative findings and postoperative chest radiographs demonstrating full lung re-expansion, as no universally accepted standardized scoring system was applied across all cases.*”
**Comment 8. The assessment of lung expansion is central to your conclusions. However, you have not described the criteria used to categorize expansion as “full” or “nearly full”. Could you clarify how you performed these assessments and whether any inter-observer variability was evaluated?**

**Response:** In our study, lung expansion was assessed primarily through postoperative chest radiographs. "Full expansion" was defined as the absence of residual pleural space or significant pneumothorax, with restoration of the lung contour to the chest wall. "Nearly full expansion" referred to cases with minimal residual pleural space without significant collapse or evidence of trapped lung. These assessments were made by the operating surgeon together with the attending radiologist. However, we acknowledge that no formal inter-observer variability analysis was performed, and the evaluation may be subject to some degree of subjective interpretation. We have clarified these definitions and acknowledged this limitation in the revised manuscript at the outcome definition part as follow;

“*Lung expansion was assessed primarily through postoperative chest radiographs. Full expansion was defined as the absence of residual pleural space or significant pneumothorax, with restoration of the lung contour to the chest wall. Nearly full expansion referred to cases with minimal residual pleural space without significant collapse or evidence of trapped lung. These assessments were made by the operating surgeon together with the attending radiologist.*”

And I added sentences in the limitation of the study as follow;

“*Another limitation is the subjective assessment of lung expansion. The criteria for "full" and "nearly full" expansion were based on the operating surgeon’s and attending radiologist’s evaluation of postoperative chest radiographs. No formal inter-observer variability analysis was conducted, which may introduce some degree of observer bias in the categorization of lung expansion*.”

**Comment 9. I believe you tend to emphasize the benefits of VATS, but open thoracotomy may remain preferable in certain scenarios. Could you discuss potential indications/advantages of open thoracotomy?**

**Response:** We agree that, despite the recognized benefits of VATS, open thoracotomy retains important roles in the management of pleural empyema and related thoracic conditions. Open thoracotomy may be preferable in cases with advanced-stage disease, dense or calcified pleural peel, extensive adhesions, or when visualization and access are limited during minimally invasive surgery. It also allows for more extensive debridement and better control in challenging anatomical situations or in patients with prior thoracic surgeries. We have now included a discussion of these potential indications and advantages for open thoracotomy in the revised manuscript, to provide a more balanced perspective as follow;

“*While VATS offers benefits, OT remains essential in select cases. It is preferred for advanced-stage disease, dense or calcified pleural peel, extensive adhesions, or when visualization is limited. OT also allows more thorough debridement and better management in complex or re-operative cases. Thus, it remains a vital option for appropriately selected patients with thoracic empyema*.”

**Comment 10. If data on recurrence, mortality, or readmission are available, consider including them. If not available, consider adding a comment on this limitation.**

**Response:** No in-hospital mortality was observed in either group of study. Unfortunately, data on recurrence, mortality, and readmission were not comprehensively available for all patients in our study, primarily due to limitations in retrospective data collection. We have now acknowledged this as a study limitation in the revised manuscript as follow;

“*Data on recurrence, mortality, and readmission were not comprehensively available for all patients. This limitation may affect the completeness of outcome assessment in our study*.”

I add “in-hospital mortality” in the result part as follow;

“*No in-hospital mortality was observed in either group*.”

**Reviewer #2: I read with great interest your article on the comparative analysis of surgical outcomes between VATS and open thoracotomy for the treatment of thoracic empyema. I would like to congratulate the authors on their results and the large sample size. However, I have several comments and recommendations that may enhance the interest of readers in your work:
Comment 1: I suggest replacing the term “empyema thoracis” with “thoracic empyema” for improved clarity and consistency with commonly used terminology.**

**Response:** We agree that “thoracic empyema” is a clearer and more widely accepted term. We have revised the manuscript accordingly for improved clarity and consistency.

**Comment 2:**  **Line 69: Please correct the syntax of the sentence, as the word “caused” appears twice.**

**Response:** We have corrected the sentence to remove the duplicate use of “caused.”
**Comment 3: You state that you only operated patients with stage III empyema. In my experience, it is often difficult to determine the exact stage of a thoracic infection preoperatively, as CT scans, blood tests, or chest drain output may not reliably reflect the presence of pleural thickening. Could you clarify how you identified the appropriate timing for surgery in confirmed stage III cases?**

**Response:** We agree that accurately determining the stage of empyema preoperatively can be challenging, as imaging and clinical assessments may not always reliably distinguish stage III disease. In our practice, the decision to proceed with surgery was based on a combination of clinical criteria—such as persistent sepsis or respiratory compromise despite appropriate medical management—radiological findings suggestive of organized empyema or pleural thickening, and intraoperative confirmation. Definitive staging was established during surgery, with stage III empyema identified by the presence of a thickened, fibrous pleural peel requiring decortication. We have clarified this process in the revised manuscript as followed;

“*Patients included in this study were those with stage III thoracic empyema who underwent surgical intervention. Due to the challenge of accurately determining empyema stage preoperatively, the decision for surgery was based on persistent sepsis or respiratory compromise despite appropriate medical therapy, in conjunction with radiological findings suggestive of organized empyema or pleural thickening. Definitive staging was established intraoperatively. Stage III empyema was defined by the presence of a thickened, fibrous pleural peel requiring decortication at surgery [5].”*

**Comment 4: It would be helpful to describe in greater detail the decortication technique used in both VATS and thoracotomy approaches, including the instruments employed, as this is central to your study.**

**Response:** We agree that a more detailed description of the decortication technique is important for clarity and reproducibility. In the revised manuscript, we have provided additional information regarding the surgical steps and instruments used for decortication in both the VATS and thoracotomy approaches.

We added sentences in the Surgical Technique of Video-Assisted Thoracoscopic Surgery (VATS) and added 3 pictures in the manuscript as follow;

*“The decortication technique performed via VATS is the same as that used in OT. We use the same instruments as in the operating room, including a sponge holder, peanut dissector sponge, and periosteum dissector, to perform decortication, as shown in Figs 2 and 3. VATS instruments may be used in deep areas where conventional instruments cannot adequately grasp the tissue, as shown in Fig 3.”*

*Fig 1. Location of skin incisions for the utility port and camera port.*

*Fig 2. Instruments used for decortication*

*Fig 3. Decortication performed using a peanut dissector sponge.*

**Comment 5: Please elaborate on the intraoperative and postoperative anesthetic management of patients. For example:
o Is the lung completely collapsed or slightly inflated to facilitate dissection?
o In some centers, patients remain intubated for 24–48 hours postoperatively to support lung re-expansion. Do you prefer early extubation? Please provide details of your protocol and rationale.**

**Response:** Thank you for your insightful comment regarding intraoperative and postoperative anesthetic management. We added information as shown below;

At “Surgical Technique of Video-Assisted Thoracoscopic Surgery (VATS)”

“*Throughout the procedure, the lung was completely collapsed to provide optimal exposure. However, intermittent inflation with low tidal volume ventilation was performed when needed to facilitate precise dissection and to prevent low tidal volume ventilation was used to prevent iatrogenic lung injury. Blunt dissection was then carried out, followed by flushing the thoracic cavity with warm saline*.”

We added new paragraph as shown below;

“Postoperative Anesthetic Management

Our institutional protocol emphasizes early extubation for both VATS and OT approaches, with the goal of extubating patients in the operating room following confirmation of stable respiratory function and hemodynamic parameters. Patients are not routinely maintained on mechanical ventilation postoperatively, except in cases of inadequate ventilation, persistent hemodynamic instability, or other perioperative complications. Early extubation is implemented to minimize the risks associated with prolonged intubation. Following surgery, patients are transferred to the recovery unit and provided with supplemental oxygen and continuous monitoring. Postoperative analgesia is achieved primarily with intravenous opioids, supplemented by intercostal nerve blocks when necessary. Chest radiographs and clinical evaluations are performed to assess lung re-expansion. If satisfactory oxygenation and lung inflation are achieved, re-intubation is not indicated and standard postoperative care is continued.”

**Comment 6: How do you assess full lung re-expansion, and do you have a defined criterion for complete decortication? These are subjective evaluations, and it is often difficult to assess them reliably via chest X-rays, CT scans, or even intraoperatively. Providing a clear definition would strengthen your methodology.
Response:** We added sentences in method part (outcome definition) as shown below;

*“Complete decortication was defined as the meticulous removal of the organized fibrous peel from visceral pleural surfaces to achieve full lung re-expansion [6]. The extent of decortication was confirmed intraoperatively by the operating surgeon through direct inspection and assessment of pulmonary re-expansion. This evaluation was based on intraoperative findings and postoperative chest radiographs demonstrating full lung re-expansion, as no universally accepted standardized scoring system was applied across all cases.* *Lung expansion was assessed primarily through postoperative chest radiographs. Full expansion was defined as the absence of residual pleural space or significant pneumothorax, with restoration of the lung contour to the chest wall. Nearly full expansion referred to cases with minimal residual pleural space without significant collapse or evidence of trapped lung. These assessments were made by the operating surgeon together with the attending radiologist.”*

And add sentences in the limitation of this study as follow;

*“Another limitation is the subjective assessment of lung expansion. The criteria for "full" and "nearly full" expansion were based on the operating surgeon’s and attending radiologist’s evaluation of postoperative chest radiographs. No formal inter-observer variability analysis was conducted, which may introduce some degree of observer bias in the categorization of lung expansion.”*

**Comment 7: Patients who underwent open thoracotomy had a significantly longer duration of symptoms, even after propensity score matching. Could this suggest that similar outcomes between thoracotomy and VATS might be due to more advanced disease in the thoracotomy group? Please address this point in the manuscript.**

**Response:** Thank you for this important observation. We acknowledge that patients in the open thoracotomy group exhibited a significantly longer duration of symptoms even after propensity score matching, which may indicate a more advanced stage of disease at the time of surgery. This difference in disease severity could potentially influence clinical outcomes. We further analyzed the data by including duration of symptoms as a variable adjusted for in the multivariable analysis model (Table 3), as shown below:

***Table 3.*** *Multivariable regression analysis for outcome variables comparing between the two groups (VATS versus OT)*

| *Outcome variables* | *All patient cohort* | | | *Propensity-matched cohort* | | |
| --- | --- | --- | --- | --- | --- | --- |
|  | *Estimate* | *95 % CI* | *p-value* | *Estimate** | *95 % CI* | *p-value* |
| *Intraoperative blood loss (ml)* | *-35.58^a^* | *-141.17, 70.12* | *0.508* | *-34.20* | *-162.89, 94.49* | *0.601* |
| *Operative time (min)* | *-6.37^a^* | *-27.17, 14.44* | *0.548* | *1.25* | *-24.42, 26.91* | *0.924* |
| *ICU stay (day)* | *1.38^a^* | *-0.68, 3.44* | *0.189* | *0.68* | *-1.56, 3.23* | *0.494* |
| *Decreased pain score* | *0.71^b^* | *-0.04, 1.46* | *0.065* | *0.45* | *-0.29, 1.18* | *0.236* |
| *Fully expanded lung at discharge date* | *1.24^c^* | *1.07, 1.45* | *0.006* | *1.21* | *1.01, 1.45* | *0.038* |
| *Fully expanded lung at 2 weeks after discharge* | *1.08^c^* | *0.95, 1.23* | *0.221* | *1.14* | *0.97, 1.34* | *0.117* |

*^a^Analyzed by multiple linear regression analysis adjusted by multi-loculation, thick pleural peel, year of surgery, and duration symptoms prior to diagnosis, and presented with mean difference (MD) with 95 % confidence intervals.*

*^b^Analyzed by repeated measurements method adjusted by age, gender, operative time, year of surgery, and duration symptoms prior to diagnosis.*

*^c^Analyzed by exponential risk regression analysis adjusted by multi-loculation, thick pleural peel, year of surgery, and duration symptoms prior to diagnosis, and presented with risk ratio (RR) with 95 % confidence intervals.*

**Adjusted for year of surgery and duration symptoms prior to diagnosis.*

We have addressed this limitation in the revised manuscript as follow;

*“Additionally, despite propensity score matching, patients in the OT group had a significantly longer duration of symptoms, suggesting more advanced disease at the time of surgery. This difference could have influenced clinical outcomes and may, in part, account for the comparable results observed between the OT and VATS groups. Therefore, residual confounding due to differences in baseline disease severity cannot be completely excluded, even though this variable was adjusted for in the multivariable analysis”*
